# Evaluating the necessity of edema-sensitive imaging in assessing fragility fractures of the pelvis: a retrospective cohort study utilizing the OF Pelvis Classification and Score

**DOI:** 10.1016/j.bas.2026.106656

**Published:** 2026-08-20

**Authors:** Mark Unthan, Felix Kohler, Florian Bürckenmeyer, Camilla Heinen, Philipp Schenk, Tobias Franiel, Bernhard W. Ullrich

**Affiliations:** a Department of Trauma, Hand and Reconstructive Surgery and Orthopedics, Jena University Hospital, Friedrich Schiller University, Am Klinikum 1, Jena, 07747, Germany; b Institute for Diagnostic and Interventional Radiology, Jena University Hospital, Friedrich Schiller University, Am Klinikum 1, Jena, 07747, Germany; c Innovationhub for Musculoskeletal Surgery Halle (IMCH), BG Klinikum Bergmannstrost Halle gGmbH, Merseburger Straße 52, Halle (Saale), 06110, Germany; d Department of Trauma and Reconstructive Surgery, BG Klinikum Bergmannstrost Halle gGmbH, Merseburger Str. 165, Halle (Saale), 06112, Germany; e Department for Trauma and Reconstructive Surgery, University Hospital Halle, Halle (Saale), 06112, Germany

**Keywords:** Pelvic ring fragility fracture, MRI, OF Pelvis Score

## Abstract

**Introduction:**

The incremental value of edema-sensitive imaging beyond CT in fragility fractures of the pelvis (FFP) remains debated.

**Research question:**

Does edema-sensitive MRI provide incremental diagnostic value beyond CT for FFP, and does this influence Osteoporotic Fracture (OF) Pelvis scoring and treatment planning?

**Materials and methods:**

In a prospective, single-center diagnostic cohort (April 2021–February 2022), adults with suspected FFP underwent pelvic CT and MRI. OF Pelvis types I–V and the OF Pelvis Score were determined; the score was calculated once with CT information and again with MRI information (retrospective). Primary outcomes were CT sensitivity and specificity for detecting clinically relevant injury (OFP III–V vs. I–II). Secondary outcomes included differences in the OF Pelvis Score and concordance of treatment recommendations.

**Results:**

Among 85 screened, 44 were analyzed (31 female, 13 male; mean age 80, range 54–94). CT showed sensitivity 86.9% (95% CI 71.9–95.5); specificity 66.7% (95% CI 29.9–92.5). MRI increased mean OF Pelvis Score (4.25 ± 3.31) versus CT obtained OF Pelvis Score (3.48 ± 3.83; p = 0.029). Despite the score shift, CT- and MRI-based treatment recommendations did not differ significantly (p = 0.317). Nine patients underwent surgery; one CT-missed unilateral sacral fracture required surgery after mobilization failure.

**Discussion and conclusion:**

CT showed high sensitivity for clinically relevant OF Pelvis III–V injuries; MRI modestly increased the OF Pelvis Score without materially changing management. A CT-first strategy for routine FFP assessment is supported, with edema-sensitive imaging reserved for diagnostic uncertainty or discordant findings.

## Highlights

• CT demonstrated high sensitivity for clinically relevant OF Pelvis III–V injuries compared with MRI.
• MRI modestly increased the OF Pelvis Score vs. CT, but the score shift didn't significantly change treatment recommendations.
• A CT-first imaging strategy for suspected fragility fractures of the pelvis is supported, with edema-sensitive MRI reserved for diagnostic uncertainty or discordant findings.

## Graphical abstract

**Figure:**
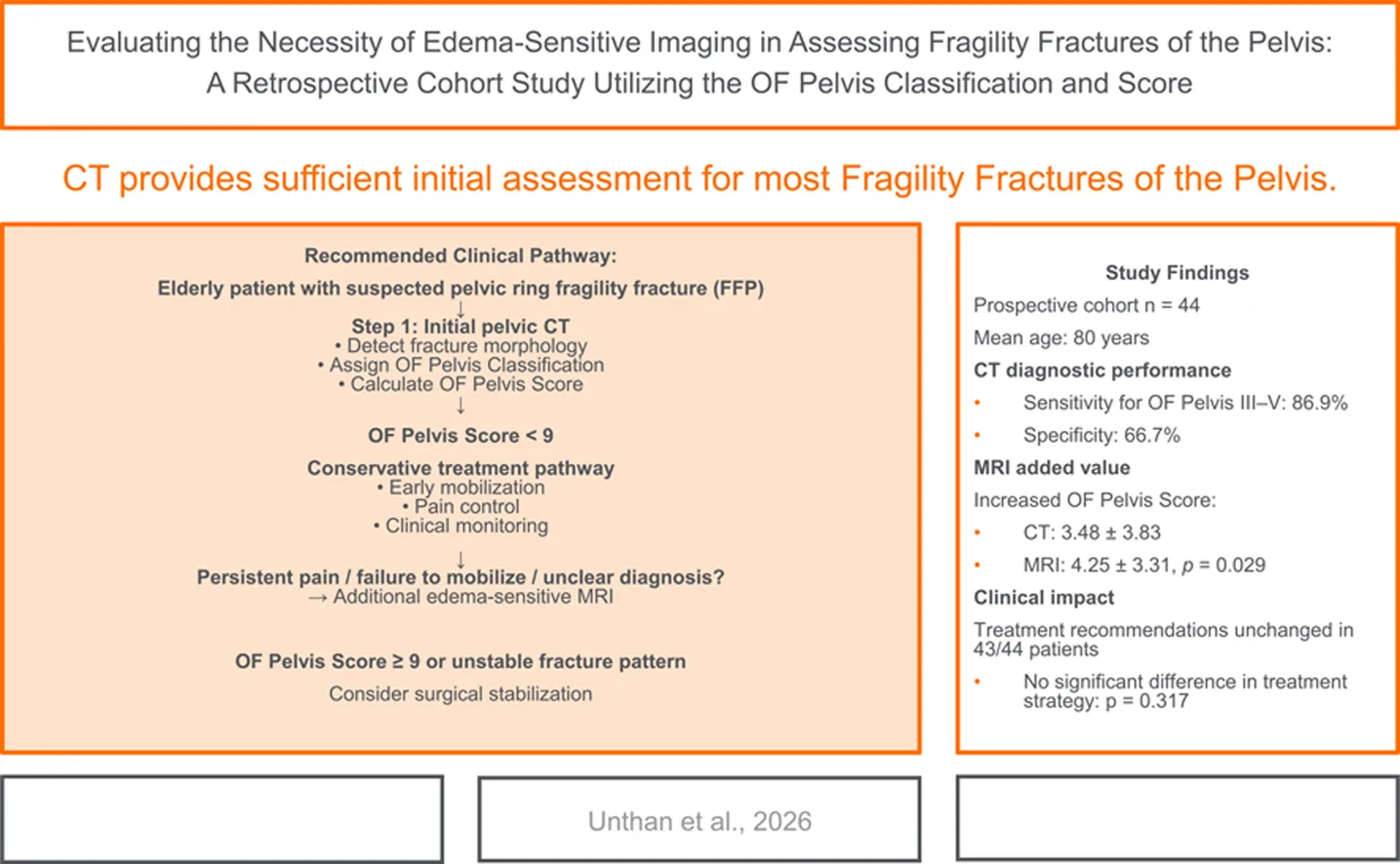

## 1 Background

Fragility fractures of the pelvis (FFP) remain a significant topic in orthopedic trauma research (Gordon et al., 2025; Gewiess et al., 2024). The vulnerable geriatric population makes accurate diagnosis and effective treatment of these injuries critical for ensuring timely and efficient care (Desmet et al., 2024; Kleeblad et al., 2023). Geriatric trauma patients require special attention due to high complication rates; however, considerations of cost-effectiveness and the optimal use of limited healthcare resources are increasingly important for trauma surgeons (Oberkircher et al., 2018; Rommens and Hofmann, 2022).

FFPs typically result from low-energy falls in elderly, often osteoporotic, patients (Bhattacharya et al., 2016). Many patients present immediately to the emergency department, while others experience delayed presentation due to progressive fractures (Mendel et al., 2021; Yamamoto et al., 2024). The alar voids are particularly vulnerable in patients with impaired bone density (Wagner et al., 2014, 2016). Those critical regions located in the alae of the Os sacrum, where bone density can decrease, comparable to nearly water, are especially susceptible. Patients often exhibit a characteristic fracture pattern through the alar voids, mostly combined with fractures of the pubic rami (Endo et al., 2025). The AO classification can be applied to categorize these predominantly lateral compression fractures, but dedicated classifications systems have been developed to better reflect the characteristics of FFPs. The FFP classification has gained widespread acceptance due to its clinical applicability and focus on fracture stability (Rommens and Hofmann, 2013). More recently, the OF Pelvis fracture classification demonstrates superior inter- and intra-rater reliability and is linked to the OF Pelvis Score, which provides treatment recommendations (Ullrich et al., 2021). The OF Pelvis Score has shown promising reliability and reproducibility in guiding clinical management (Spiegl et al., 2025, 2026).

Diagnosing FFPs poses a challenge (Lyders et al., 2010). Plain radiographs have limited sensitivity compared to CT scans. Both national and international guidelines recommend native pelvic CT scans for diagnosis or exclusion of fractures (Kleeblad et al., 2023; Deutsche Gesellschaft für Orthopädie und, 2023). MRI is highly sensitive and useful for detecting edema-sensitive sequelae associated with FFPs (Cabarrus et al., 2008). Advanced imaging modalities such as spectral CT or dual-energy CT can also generate edema-sensitive sequences (Unthan et al., 2024; Guggenberger et al., 2012). Retrospective studies have demonstrated MRI's superior diagnostic accuracy in identifying FFPs, particularly sacral fractures, whether unilateral or bilateral (Graul et al., 2020).

Treatment options for pelvic ring fragility fractures vary (Kleeblad et al., 2023). The OF Pelvis Score suggests conservative management with full weight-bearing and adequate analgesia for lower scores, while higher scores indicate the need for surgical intervention. Well established minimally invasive procedures (Kleeblad et al., 2023), such as sacroiliac screw osteosynthesis, either tricortical or hexacortical, spinopelvic fixation, or modern implants designed for minimally invasive application (Marintschev and Hofmann, 2023), are tailored to the surgeon's needs. While the most effective surgical approach for FFPs remains an area for further multicenter research, recent developments focus on bilateral fracture fixation with newer implants.

There is currently no conclusive evidence regarding the necessity of pelvic MRI in diagnosing FFPs, despite its recommendation in national and international guidelines (Kleeblad et al., 2023; Deutsche Gesellschaft für Orthopädie und, 2023). Our aim was to evaluate the role of MRI in the diagnosis of pelvic ring insufficiency fractures within routine clinical practice.

## 2 Methods

### 2.1 Aim of the study

Our objective was to evaluate the influence of edema sensitive MRI images in routine clinical practice in the diagnosis of pelvic ring insufficiency fractures and the consequent influence on treatment decisions, taking into account the OF Pelvis Classification and the OF Pelvis Score.

### 2.2 Study design & participants

In a single-center study, patients with suspected FFP were prospectively enrolled consecutively between April 2021 and February 2022. All patients underwent native CT and MRI of the pelvis. Inclusion criteria were evidence of Osteopenia or Osteoporosis, decreased Hounsfield units (<130HU) in L5 as an indicator of reduced bone density on the CT scan (Ullrich et al., 2022), Patients with high-energy fractures, known preexisting bone marrow disease, and any contraindications for MRI (such as pacemakers or claustrophobia) were excluded. All patients provided written consent.

### 2.3 Imaging modalities

CT images were obtained using a GE Revolution CT scanner. During the study period, three different MRI scanners were employed for diagnostic purposes in our clinic: Siemens Magnetom Sola, Siemens Magnetom Vida, and Siemens Magnetom Aera. The assessment of edema was conducted using fluid-sensitive fat-saturated sequences, including STIR/TIRM and PDw fatsat, in both axial and coronal planes, alongside additional axial and coronal T1-weighted sequences. The CT and MRI images of the pelvis were pseudonymized and devoid of any clinical information. A radiologist specialized in musculoskeletal imaging graded the images in a randomized order. Findings for each image series were documented on standardized reporting sheets.

### 2.4 OF Classification and OF Pelvis Score

The fractures were classified based on the OF Pelvis Classification. Classification was established across both CT and MRI imaging. Specifically, OF Pelvis type I injuries are characterized solely by pelvic ring edema. Type II injuries involve isolated anterior pelvic ring lesions, while type III refers to unilateral sacral fractures, which may occur with or without associated anterior ring lesions. Type IV injuries are characterized by bilateral sacral fractures, with or without anterior ring lesions, and type V fractures include iliac or sacroiliac fractures, which may also occur with or without anterior ring lesions (Ullrich et al., 2021).

Retrospectively, the OF Pelvis Score was calculated during the first three days following hospital admission, utilizing available data from patient records (Spiegl et al., 2025, 2026). The score is based on several criteria, including fracture morphology (OF Pelvis Classification), mobilization status, pain (assessed using the Numerical Rating Scale (NRS)), neurological assessment related to the fracture, overall health status, and relevant modifications, as outlined in Table 1. A cumulative score ranging from 0 to 7 indicates that non-operative treatment is recommended. A score of 8 denotes an ambiguous situation that requires individual clinical judgment, while a score of 9 or higher suggests that surgical intervention may be warranted. The score was calculated once with the information of the MRI and once without.

**Table 1:** OF Pelvis Score.

**Fracture morphology**	OF Pelvis Classification 1–5 (2–10)
**Mobilization**	immobile (2), very limited (0), sufficiently mobile (−2)
**Pain (VAS)**	≥5 (1), <5 (−1)
**Neurology**	deficit present (1)
**State of health (ASA, mFI, anticoagulant medication)**	ASA >3, mFI ≥2, Anticoagulant medication: yes (−1 each, max −2)
**Modifying factors max 1 point:** M1: L5 transverse fracture on CT (1) M2: fracture dislocation (1) M3: fracture edema at another location (MRI) (1)	
**Treatment recommendation based on total points:** 0–7: Conservative treatment 8: Relative indication for surgery 9–15: Surgery recommended	
ASA: American Society of Anesthesiologists score; mFI: modified Frailty Index; VAS: visual analog scale. If a characteristic cannot be evaluated or is unknown, it is assigned 0 points.	

### 2.5 Treatment

Patient treatment followed national guidelines (Deutsche Gesellschaft für Orthopädie und, 2023). Early mobilization under the supervision of a trained physical therapist is initiated, to reduce or avoid bed rest.

Pain and mobilization status are evaluated and documented daily. Pain is assessed using the Numeric Rating Scale (NRS), ranging from 0 (no pain) to 10 (worst imaginable pain). The physical therapist employs various walking aids, including forearm crutches, rigid walking frames, early mobility walkers with padded armrests (EVA), and wheeled walkers. Pain medication is administered according to WHO recommendations. In our routine protocol, Metamizole is used as a first-line analgesic. If necessary, second-line treatment involves Tilidine with Naloxone, and third-line analgesia typically consists of Oxycodone with Naloxone.

Operative treatment varies as described in the literature. In our practice, we use hexacortical cannulated 7.3 mm sacroiliac (SI) screws (DePuy Synthes, Norderstedt, Germany) or atypical screws tailored to sacral morphology. Depending on the clinical presentation, screws are augmented with trauma cement. The implant of choice is the SACRONAIL® (Signus Medical GmbH, Alzenau, Germany). This minimally invasive, bilateral fixed-angle locking device is implanted using a minimally invasive approach and is considered a safe treatment option (Marintschev and Hofmann, 2023). Treatment of pubic ramus fractures is rare and is only considered in highly unstable cases.

### 2.6 Analysis

Data were collected using Microsoft Excel and subsequently exported to SPSS (IBM SPSS Statistics for Windows, Version 29.0, Armonk, NY: IBM Corp) for statistical analysis. Diagnostic accuracy for subgroups defined by the presence of dorsal fracture patterns was assessed using contingency tables. The McNemar test was employed to compare the presence of a dorsal fracture pattern to evaluate diagnostic accuracy of the CT compared to the MRI as referred as Gold standard. The normality distribution of the OF Pelvis Score was checked with the Kolmogorov-Smirnov test. Differences in the OF Pelvis Scores were analyzed using the Wilcoxon signed-rank test. Treatment recommendations were categorized as non-operative, equivocal, or operative based on the OF Pelvis Score. Since treatment recommendation represents a paired ordinal variable, agreement between CT- and MRI-based recommendations was assessed using the test of marginal homogeneity.

## 3 Results

**Fig. 1:**
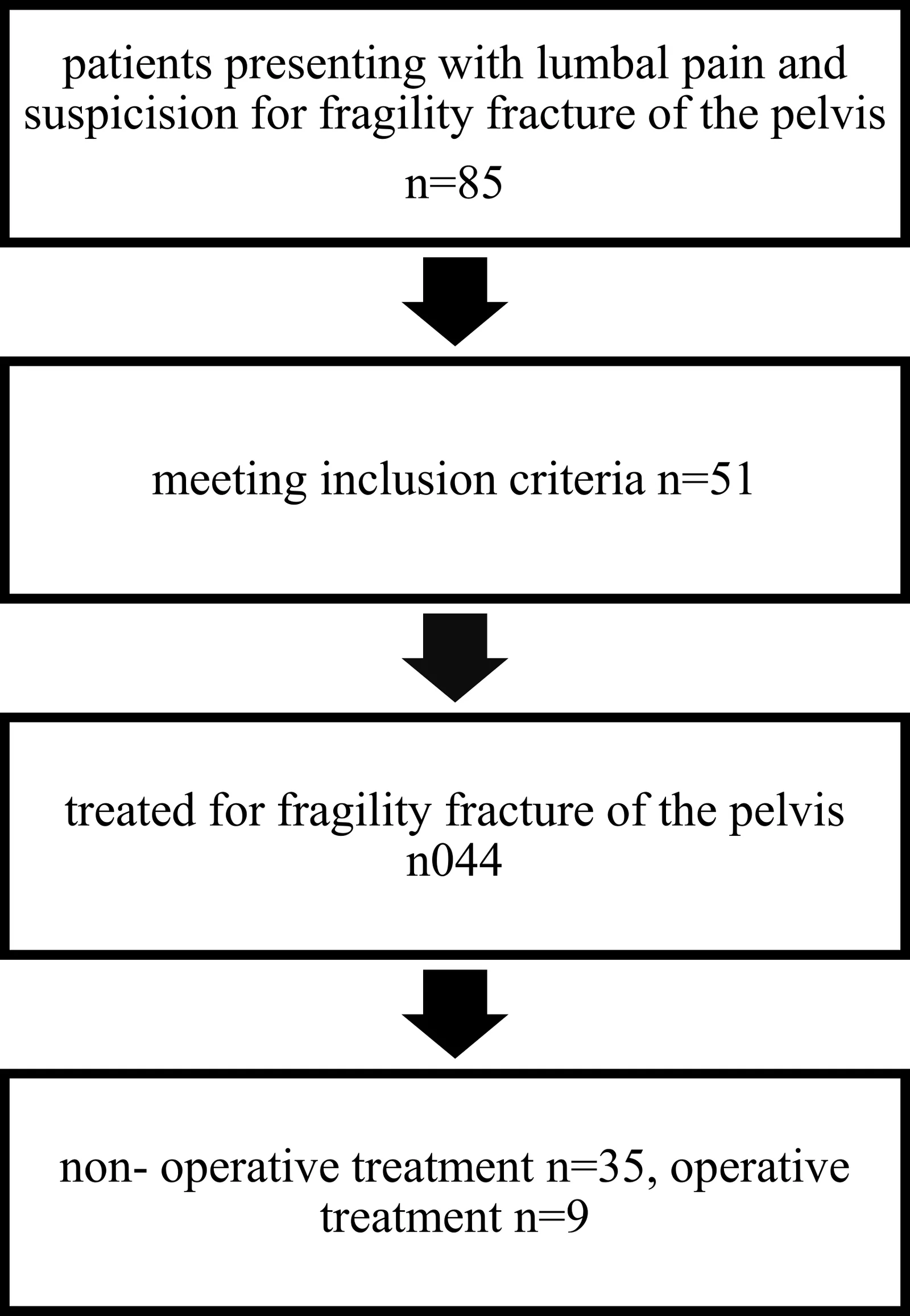
Flow of participants.

Among 85 screened patients, 51 met the inclusion criteria (Fig. 1). Seven patients were excluded due to fracture characteristics identified on CT and MRI, resulting in their omission from the analysis. The final cohort consisted of 44 patients, including 13 men and 31 women, with a mean age of 80 years (range: 54–94 years). The L5 vertebral Hounsfield units (HU) averaged 68 ± 30 HU (range: 10–106 HU). All patients underwent spectral CT upon admission, followed by pelvic MRI after an average of 2 ± 3 days (range: 0–13 days).

MRI assessment classified the fractures according to the OF Pelvis Classification as follows: OF Pelvis I in 1 patient, OF Pelvis II in 4, OF Pelvis III in 26, OF Pelvis IV in 11, and OF Pelvis V in 1 (Table 2). CT imaging resulted in reclassification to a lower OF Pelvis grade in three cases: two bilateral sacral fractures initially classified as OF Pelvis IV were downgraded to unilateral fractures (OF Pelvis III), and one posterior pelvic ring fracture was reclassified as an isolated pubic rami fracture (OF Pelvis II). Additionally, one posterior pelvic ring fracture was initially missed (false negative). Conversely, five fractures were initially overlooked on conventional CT, including four unilateral sacral fractures and one bilateral fracture. Notably, six cases were reclassified from unilateral to bilateral sacral fractures based on further imaging.

**Table 2:** OF Pelvis Classification based on CT or MRI imaging.

OF Pelvis Classification		MRI-based						Total
		0	1	2	3	4	5	
CT-based	0	0	1	0	4	1	0	6
	1	0	0	0	0	0	0	0
	2	0	0	3	0	0	0	3
	3	0	0	1	20	6	0	27
	4	1	0	0	2	4	0	7
	5	0	0	0	0	0	1	1
Total		1	1	4	26	11	1	44

### 3.1 Diagnostic accuracy of CT

Compared to the reference standard of pelvic MRI, the sensitivity of pelvic CT was 86.9% (95% CI: 71.9%–95.5%), and the specificity was 66.6% (95% CI: 54.5%–98.0%; p = 0.453), as determined by the McNemar test when comparing the presence of Pelvis Classifications 0–II versus III–V (see Table 3).

**Table 3:** Diagnostic accuracy of the conventional CT pelvis (index test) compared to MRI pelvis (reference test).

		MRI pelvis		
		OF Pelvis 0-II	OF Pelvis III-V	n
CT	OF Pelvis 0-II	4	5	9
	OF Pelvis III-V	2	33	35
	N	6	38	44

### 3.2 OF Pelvis Score calculation

The OF Pelvis Score was calculated between days 3 and 4 post-injury (mean: 3.6; range: 1–10; standard deviation: 2.0). Due to the non-Gaussian distribution, the Wilcoxon signed-rank test was employed, revealing a significant difference in scores (p = 0.029). The mean OF Pelvis Score calculated using CT was 3.5 (range: −4 to 12; SD: 3.3), while the MRI-based score averaged 4.3 (range: −3 to 12; SD: 3.). Applying the cutoff values recommended by the original score authors, the discrepancies between the two modalities appeared subtle. There was no statistically significant difference between the treatment recommendation from the CT and MRI based OF Pelvis Score p = 0.317.

Nine patients underwent surgical intervention, primarily utilizing SACRONAIL® in five cases, sacroiliac screws in three, and open reduction in one case of transiliac fracture. Among the 44 patients treated for fragility fractures, only one patient with an initially undiagnosed unilateral pelvic fracture on CT subsequently underwent surgery, highlighting a discrepancy. Further review of this patient's history revealed a complex case: although initial CT did not suggest a typical fracture pattern, clinical examination demonstrated tenderness over the lateral sacrum and a pain NRS of 10/10. MRI obtained within one day confirmed an OF Pelvis III fracture. The patient was initially managed non-operatively to avoid surgical risks in a multimorbid context. However, due to unsuccessful mobilization, surgical intervention was ultimately performed, resulting in acceptable mobility at discharge.

The patient who was treated operatively despite an OF Pelvis score of 7 without radiographic evidence of fracture displacement reported prolonged dissatisfaction with conservative management. Following surgical treatment, the patient reported a satisfactory clinical outcome.

## 4 Discussion

The diagnosis of pelvic ring fragility fractures remains a challenging task. A common requirement is computed tomography (CT) evaluation to assess fracture morphology (Kleeblad et al., 2023). The role of edema-sensitive imaging modalities in fully characterizing the extent of lateral compression fractures is still under investigation (Unthan et al., 2024; Palm et al., 2020). Concerns have been expressed regarding the potential for edema-sensitive imaging to result in overtreatment. A recent analysis conducted by Lang et al. provides retrospective data that may challenge this hypothesis (Lang et al., 2021). Promising data have been presented by Palm et al., who utilized SPECTRAL or dual-energy CT techniques to generate edema-sensitive images based on calcium-free images derived from differential absorption levels during SPECTRAL or dual-energy CT scans (Palm et al., 2020). However, our recent work demonstrated no significant difference in diagnostic accuracy between these methods (Unthan et al., 2024). Magnetic resonance imaging (MRI) is still considered the gold standard; nonetheless, further advancements in CT imaging could challenge its primacy (Kleeblad et al., 2023).

Retrospective analyses, such as those published by Graul et al., suggest that edema-sensitive imaging can provide critical information when surgical intervention is considered (Graul et al., 2020). National guidelines emphasize the role of MRI in this context (Deutsche Gesellschaft für Orthopädie und, 2023). This contrasts with our clinical experience, where management of fragility fractures based solely on CT findings has yielded satisfactory outcomes in most cases. The widely used Rommens classification system for FFP has been instrumental in categorizing these injuries (Endo et al., 2025). For our imaging research, we adopted the OF Pelvis Classification, a previously introduced system tailored for FFP fractures (Ullrich et al., 2021). The OF Pelvis Classification has demonstrated higher inter- and intra-rater reliability compared to the Rommens system and incorporates information from edema-sensitive imaging (Pieroh et al., 2019).

Most of the existing data were retrospectively collected; however, our study was designed to include consecutive cases over a defined period. The diagnostic accuracy of CT in our cohort was 86.9%, exceeding previously published figures. Advances in CT technology, including modified sequences and increased experience among musculoskeletal radiologists, likely contribute to this improvement. The sample size was determined based on a comprehensive review of the literature Although MRI significantly influenced fracture classification and the OF Pelvis Score, this study was not designed or powered to detect differences in treatment recommendations. A sensitivity analysis demonstrated that the available sample size was sufficient to detect only moderate paired effects, whereas smaller effects may have remained undetected. Therefore, subtle changes in treatment strategy cannot be excluded. The small sample size was primarily due to resource constraints related to MRI availability. A larger cohort may provide more precise estimates, but our data are prospectively and consecutively collected. To support robust recommendations for national and international guidelines and clinical practice, further studies with larger sample sizes and complementary study designs are warranted.

Another limitation is the timing of MRI acquisition. Although we aimed for minimal delay, some outliers occurred. Imaging conducted 5 to 7 days after an injury, using either CT or MRI, can show signs of fracture progression (Mendel et al., 2021). This observation aligns with our experiences in repeated CT scans, indicating that changes in the fracture status may become more apparent within this timeframe. The OF Pelvis Scores were calculated retrospectively, as this scoring system was neither published nor validated at the time of study initiation. The OF Pelvis Score offers direct treatment recommendations, unlike the FFP classification, which complicates treatment decisions based solely on classification. Even retrospectively in this cohort, there was a high concordance between the OF Pelvis Scores and actual clinical therapy. The OF Pelvis Score emphasizes clinical parameters such as mobility, fragility, and pain, with high sensitivity attributed to clinical diagnostics, particularly the presence of pain in the Os sacrum. Our analysis demonstrated a strong correlation between the score and the clinical decisions made. Although the score results significantly differed when derived from MRI versus CT (OF Pelvis Scores), the recommended therapy was consistent in both assessments, except in one case where bilateral fracture was overlooked in the blind analysis. In this specific instance, the typical clinical presentation kept the suspicion for a fragility fracture elevated (Nüchtern et al., 2015). The relevance of edema-sensitive imaging may also be influenced by surgical strategy. Currently, there is no definitive evidence supporting the necessity of bilateral sacroiliac or sacropelvic stabilization, although many of the newer, tailored implants suggest a prophylactic, bilateral stabilization approach (Gordon et al., 2025). When considering unilateral sacroiliac stabilization, especially in cases of dysmorphic sacral anatomy, a fracture progression should be carefully monitored (Mendel et al., 2021). Based on our data, the recommendation for edema-sensitive imaging can be reduced to a clinical decision-making tool in cases of diagnostic uncertainty. We cannot provide evidence that clinical practice is currently guided by edema-sensitive imaging or that patients derive a significant benefit from it.

## 5 Conclusions

Our data suggests that careful clinical examination combined with the OF Pelvis Classification and the OF Pelvis Score provides reliable guidance for managing pelvic fragility fractures. Although MRI tended to increase the OF Pelvis Score, this did not result in statistically significant differences in therapy recommendations. These findings support the continued use of clinical assessment and CT-based classification in routine practice, while edema-sensitive imaging may be reserved for cases with diagnostic uncertainty until further evidence.

## Ethics approval and consent to participate

The study was approved by the Ethics Committee Jena 2124-MV.

## Consent for publication

All patients in this study gave consent to the processing of personal data for scientific purposes.

## Availability of data and materials

The original contributions presented in the study are included in the article, further inquiries can be directed to the corresponding author.

## Funding

This research did not receive any specific grant from funding agencies in the public, commercial, or not-for-profit sectors.

## Declaration of competing interests

The authors declare that they have no known competing financial interests or personal relationships that could have appeared to influence the work reported in this paper.

## List of abbreviations

FFP Fragility Fractures of the Pelvis.

HU Hounsfield Unit

mFI modified Frailty Index.

NRS Numeric Rating Scale.

OF Osteoporotic Fractures.

SI sacroiliac.
